# Early experience of an infectious and tropical diseases unit during the coronavirus disease (COVID-19) pandemic, Florence, Italy, February to March 2020

**DOI:** 10.2807/1560-7917.ES.2020.25.17.2000556

**Published:** 2020-04-30

**Authors:** Filippo Lagi, Matteo Piccica, Lucia Graziani, Iacopo Vellere, Annarita Botta, Marta Tilli, Letizia Ottino, Beatrice Borchi, Marco Pozzi, Filippo Bartalesi, Jessica Mencarini, Michele Spinicci, Lorenzo Zammarchi, Filippo Pieralli, Giovanni Zagli, Carlo Nozzoli, Stefano Romagnoli, Alessandro Bartoloni, the COCORA working group

**Affiliations:** 1 Department of Experimental and Clinical Medicine, University of Florence, Florence, Italy; 2 Infectious and Tropical Diseases Unit, Azienda Ospedaliero-Universitaria Careggi, Florence, Italy; 3 Intermediate Care Unit, Azienda Ospedaliero-Universitaria Careggi, Florence, Italy; 4 Department of Anesthesiology and Intensive Care, Azienda Ospedaliero-Universitaria Careggi, Florence, Italy; 5 Department of Internal Medicine, Azienda Ospedaliero-Universitaria Careggi, Florence, Italy; 6 Health Sciences Department, Section of Anesthesiology, Intensive Care and Pain Medicine - University of Florence, Florence, Italy; 7 The members of the COCORA working group are listed at the end of the article

**Keywords:** COVID-19, Cohort, ICU, Italy, Florence, pandemic, SARS-CoV-2

## Abstract

We analysed the first 84 coronavirus disease (COVID-19) patients hospitalised in an infectious and tropical disease unit in Florence, Italy, over 30 days after the start of the COVID-19 outbreak in Italy. A 12% reduction in the rate of intensive care unit transfer was observed after the implementation of intensity care measures in the regular ward such as increasing the nurse/patient ratio, presence of critical care physicians and using high flow nasal cannulae oxygenation.

Since the first case reported on 21 February 2020 in Lombardia region, Italy has become one of the countries most affected by the coronavirus disease (COVID-19) pandemic, counting more than 197,000 cases and more than 26,000 fatalities by 27 April. At the end of April, Italy has the highest numbers of confirmed deaths in Europe [1,2].

We present our first-month experience of the COVID-19 pandemic in Italy at the Infectious and Tropical Disease Unit (ITD) of the University Hospital in Florence, Tuscany. We aimed to describe clinical and demographic characteristics of hospitalised patients with COVID-19 as well as differences between hospitalised patients not transferred to the intensive care unit (ICU) and hospitalised patients later transferred to the ICU. Finally, we analysed the incidence rate of ICU transfers before and after the implementation of additional intensity care measures on our regular ward.

## Construction of the cohort and data analysis

We enrolled all laboratory-confirmed COVID-19 cases who were hospitalised in the ITD ward between 25 February and 26 March 2020. A laboratory-confirmed case was a symptomatic patient with positive result on a reverse-transcriptase (RT)-PCR assay. Demographic and clinical data were collected from electronic medical hospital records. ICU transfer was the primary outcome. Categorical variables were analysed by chi-squared or Fisher's exact test and continuous variables by the Kruskal-Wallis test. Poisson regression model was used for regression analysis. Cases were followed-up from the first day of hospitalisation to ICU transfer or hospital discharge or death. Cases still hospitalised were censored on 3 April.

Data collection was approved by the local Ethics Committee (17104_oss). The study was performed in accordance with the ethical principles of the Declaration of Helsinki and with the International Conference on Harmonization Good Clinical Practice guidelines.

## Rising intensity of care: pre- and post-intervention

Since the beginning of the COVID-19 outbreak in Italy, the intensity care level in the ITD was gradually increased. Under standard operations, hereafter referred to as ‘pre-intervention’, our ward has a nurse-patient ratio of 1:10. Thirteen days after hospitalisation of the first COVID-19 case, hereafter referred to as ‘post-intervention’, the nurse patient ratio was enhanced to 1:6, a stable team of critical care physicians was formed, and advanced ventilation support like high flow nasal cannulae oxygenation became available on the ward.

## Cohort description

Overall, we included 84 patients: 16 (19.0%) were admitted in ICU, while 68 (81.0%) did not (N-ICU). All ICU patients underwent invasive mechanical ventilation. At the data cut-off date, eight had died (9.5%), 43 were discharged (67.9%), and 19 (22.6%) were still hospitalised (six in our unit and 13 in ICU). The median age of cases was 62 years (interquartile range (IQR): 51–72), 55 (65.5%) of cases were males and 49 (58.3%) had at least one comorbidity. The comorbidities or conditions mainly associated with ICU transfer were having chronic obstructive pulmonary disease (COPD) (p = 0.016) and presenting with low Horowitz index (PaO2/FiO2 ratio) at admission (p = 0.0048). All ICU cases had radiologically confirmed pneumonia. Clinical and demographic characteristics are summarised in Table 1.

**Table 1:** Clinical and demographic characteristics at admission of hospitalised COVID-19 cases at the infectious and tropical diseases unit of the University Hospital, Florence, Italy, 25 February–26 March 2020 (n = 84)

Characteristics	Total (n = 84)		Not ICU transferred (n = 68)		ICU transferred (n = 16)		p value
	n	% or (IQR)	n	% or (IQR)	n	% or (IQR)	
Age and sex							
Age, median (years)	62	(51–72)	62	(50–72)	67	(58–71)	0.2919
Male	55	65.5	41	60.3	14	87.5	0.045^a^
Female	29	34.5	27	39.7	2	12.5	
Smoking							
Former smoker	19	22.6	12	17.6	7	43.8	0.025^a^
Smoker	6	7.1	5	7.3	1	6.2	
Non-smoker	46	54.8	42	61.8	4	25.0	
Unknown	13	15.5	9	13.2	4	25.0	
Underlying conditions							
Diabetes mellitus	12	14.3	8	11.8	4	25.0	0.230^b^
COPD	5	5.6	2	2.9	3	18.8	0.045^a^
Coronary heart disease	12	14.3	7	10.3	5	31.3	0.046^a^
Hypertension	31	36.9	26	38.2	5	31.3	0.602^b^
Chronic hepatitis B	2	2.4	1	1.5	1	6.3	0.347^a^
Cerebrovascular disease	2	2.4	2	2.9	0	0.0	NA
Former or still oncologic diseases	7	8.3	6	8.8	1	6.3	0.738^a^
Chronic renal failure	3	3.6	3	4.4	0	0.0	NA
Medication at admission							
Prior therapy with ACE inhibitor	15	17.9	12	17.6	3	18.9	1.000^a^
Prior therapy with sartan	11	13.1	9	13.2	2	12.5	1.000^a^
Median of days from the onset of symptoms to the nasal swab	6.5	(4–9)	7	(5–9)	5	(3–7.5)	0.0688
History of contact with a confirmed or suspected case in the previous 14 days	16	19.1	13	24.5	3	9.7	0.149^a^
Horowitz index at the baseline, median	324	(281–360)	332	(300–364)	280	(234–316)	0.0048
Radiologically confirmed pneumonia	73	86.9	57	83.8	16	100.0	0.084^b^
Medication during hospitalisation							
Empiric antibiotic therapy	44	52.4	30	44.1	14	87.5	0.002^b^
Protease inhibitors	72	85.7	57	83.8	15	93.8	0.307^b^
Hydroxychloroquine	71	84.5	55	80.8	16	100.0	0.057^b^
High Flow with Nasal Cannula > 24h	7	10.3	1	6.25	8	9.5	NA
Outcome at 3 April (data cut off)							
Death	8	9.5	5	7.4	3	18.8	NA
Discharged	57	67.9	57	83.8	0	0.0	
Still hospitalised	19	22.6	6	8.8	13	81.3	

ACE: angiotensin converting enzyme; COVID-19: coronavirus disease; COPD: chronic obstructive pulmonary disease; ICU: intensive care unit; IQR: interquartile range; NA: not applicable.

^a^ Fisher’s exact test.

^b^ chi square test.

Additionally, two women, both in the second trimester of pregnancy, were hospitalised.

Corticosteroid treatment was only prescribed in N-ICU patients (n = 14; 20.6%) in the post-intervention period. Overall, three patients received tocilizumab: two in N-ICU and one in ICU. Four patients received remdesivir (5.9%), all in N-ICU.

### Clinical and laboratory characteristics

Among laboratory parameters at the admission to the ward, we found a higher median of neutrophils and procalcitonin in N-ICU patients. Moreover, a higher median of C-reactive protein was observed in ICU patients compared with N-ICU (126 mg/L (IQR 72–209) and 44 mg/L (IQR 19–71) respectively; p < 0.0001). Although at the admission interleukin-6 was only available for 50 of 84 patients, we did not observe any difference in the two groups. (Table 2)

**Table 2:** Laboratory parameters at the admission of hospitalised COVID-19 cases at the infectious and tropical diseases unit of the University Hospital, Florence, Italy, 25 February–26 March 2020 (n = 84)

Parameters	Reference values	Total n = 84		Not ICU transferred n = 68		ICU transferred n = 16		p value
		Median	IQR	Median	IQR	Median	IQR	
White blood cells x 10^9^/L, median	4.00–10.00	5.2	4.03–7.5	4.9	4.0–5.9	8.3	5.1–11.7	0.0028
Neutrophils x 10^9^/L	1.50–7.50	3.7	2.8–5.5	3.4	2.6–4.9	6.0	4.2–10.2	0.0004
Lymphocytes 10^9^/L	0.50–5.00	0.81	0.6–1.3	0.83	0.7–1.31	0.5	0.4–0.9	0.0209
Platlets	140–440	175	145–226	174	145–219	195	139–255	0.6442
Haemoglobin g/dl	14.0–18.0	13.7	11.4–14.8	13.7	12.6–14.9	13.6	11.3–14.6	0.2364
ALT (U/L)	10–50	24	16–44	24	15–43	23	18–54	0.7074
Creatinine (mg/dL)	0.70–1.20	0.9	0.8–1.1	0.9	0.7–1.0	1.0	0.8–1.1	0.2389
Sodium (mEq/L)	135–145	138	136–140	138	136–140	137	134–139	0.3903
Potassium (mEq/L)	3.5–5.1	4	3.7–4.3	4	3.7–4.3	3.9	3.6–4.3	0.5742
Procalcitonin (ng/mL)	< 0.5	0.12	0.07–0.23	0.09	0.06–0.16	0.29	0.17–2.07	< 0.0001
LDH (U/L)	135–225	251	199–323	239	195–284	325	259–407	0.0014
IL-6 (PG/ML)^a^	0.0–10.0	18.2	9.1–33.9^a^	18.1	7.7–43.1^a^	18.2	9.3–26.4^a^	0.9347
C-reactive protein (mg/L)	< 5	53	24–103	44	19–71	126	72–209	< 0.0001

ALT: alanine aminotransferase; COVID-19: coronavirus disease; ICU: intensive care unit; IL: interleukin; IQR: interquartile range; LDH: lactate dehydrogenase.

^a^ Available for 50 patients: 39 not transferred in ICU, 11 transferred in ICU.

We did not find any particular variation in clinical presentation between patients transferred or not to ICU, except for diarrhoea which was more common in the N-ICU group (Table 3). Notably, three patients had an atypical presentation without fever: two syncopes, one abdominal pain. Data on self-reported dysgeusia and anosmia were available only for 48 N-ICU of 68 cases, and reported by 54.1% and 35.4%, respectively.

**Table 3:** Symptoms of hospitalised COVID-19 cases at admission to the infectious and tropical diseases unit of University Hospital, Florence, Italy, 25 February–26 March 2020 (n = 84)

Symptoms	Total (n = 84)		Not ICU transferred (n = 68)		ICU transferred (n = 16)		p value
	n	%	n	%	n	%	
Fever >38° C	73	86.9	59	86.8	14	–	0.937^c^
Cough	59	70.2	50	73.5	9	56.2	0.174^c^
Dysgeusia^a^	26	54.1^a^	26	54.1^a^	NA	–	–
Dyspnoea	25	29.8	18	26.5	7	43.8	0.174^c^
Anosmia^a^	17	35.4^a^	17	35.4^a^	NA	–	–
Asthenia	19	22.6	16	23.5	3	18.7	1.000^b^
Diarrhoea	16	19.0	16	23.5	0	0.0	–
Myalgia/arthralgia	10	11.9	9	13.2	1	6.3	0.679^b^
Sputum	9	10.7	8	11.8	1	6.3	1.000^b^
Headache	7	8.3	7	10.3	0	0.0	–
Nausea/vomit	7	8.3	7	10.3	0	0.0	–
Sore throat	5	5.9	5	7.3	0	0.0	–
Nasal congestion	4	4.8	4	5.8	0	0.0	–
Rash	2	2.4	2	2.9	0	0.0	–

COVID-19: coronavirus disease; ICU: intensive care unit; NA: not available.

^a^ Available for 48 of 68 patients.

^b^ Fisher’s exact test.

^c^ chi square test.

### Clinical characteristics in the pre-intervention and post-intervention period

We observed no difference in age, having COPD, coronary heart disease, chronic hepatitis B, cerebrovascular disease, oncologic illness, chronic renal failure, Horowitz index at admission between COVID-19 cases during the pre-intervention or post-intervention phase. The percentage of patients with diabetes mellitus and hypertension was higher at pre-intervention compared with post-intervention (25.8% vs 7.6% and 58.1% vs 24.5% respectively) (Table 4).

**Table 4:** Clinical characteristics at admission of COVID-19 cases hospitalised before and after the implementation of additional intensive care measures at the infectious and tropical diseases unit of University Hospital, Florence, Italy, 25 February–26 March 2020 (n = 84)

Clinical characteristics	Before implementation n = 31		After implementation n = 53		p value
	n	% or (IQR)	n	% or (IQR)	
Age and sex					
Age, median (years)	64	(58–72)	59	(50–51)	0.1822
Male	19	61.3	36	67.9	0.537^b^
Female	12	38.7	17	32.1	
Smoking					
Former smoker Smoker Non-smoker Missing	11 2 16 2	35.5 6.5 51.6 6.5	8 4 30 11	15.1 7.6 56.6 20.7	0.100^a^
Underlying conditions					
Diabetes mellitus	8	25.8	4	7.6	0.028^a^
COPD	2	6.5	3	5.7	1.000^a^
Coronary heart disease	5	16.1	7	13.2	0.712^b^
Hypertension	18	58.1	13	24.5	0.002^b^
Chronic hepatitis B	1	3.2	1	1.9	1.000^a^
Cerebrovascular disease	1	3.2	1	3.2	1.000^a^
Former or still oncologic diseases	3	9.7	4	7.6	0.705^a^
Chronic renal failure	1	3.2	2	3.8	1.000^a^
Horowitz index at the baseline, median	306	(276–347)	328	(295–366)	0.1029
C-reactive Protein (mg/L), median (Norm: < 5 mg/L)	50	(25–82)	64	(17–133)	0.4169

COPD: chronic obstructive pulmonary disease; COVID-19: coronavirus disease; IQR: interquartile range.

^a^ Fisher’s exact test.

^b^chi square test.

In the pre- and post-intervention period, nine (29.0%) and seven (13.2%) patients were transferred to ICU. The overall rate of ICU transfers in our unit was 2.64 (95% confidence interval (CI): 1.62–4.31) per 100 patient days. The rate of ICU transfers in the post-intervention period compared with the pre-intervention period markedly decreased from 6.30 per 100 patient days, (95% CI: 3.00–13.22) to 1.82 patient days, (95% CI: 0.94–3.50). The unadjusted rate ratio (RR) for transfers for the post-intervention period was 0.28 (95% CI: 0.10–0.75; p = 0.0070). After adjustment for sex, age, Horowitz index at baseline, diabetes mellitus, hypertension, COPD, coronary heart disease, the RR for the post-intervention period was 0.88 (95% CI: 0.82–0.95; p = 0.002).

## Discussion

The majority of cases hospitalised at ICT during the first 30 days of the COVID-19 pandemic were males older than 60 years of age. More than half of them had at least one comorbidity of which hypertension, coronary heart disease, diabetes mellitus were most frequent, oncologic illness former or still, COPD, chronic renal failure, chronic hepatitis B and cerebrovascular disease were also present in a notable number of patients. We observed that the limited number of cases for which information was available, self-reported olfactory and/or taste disorders in 35% and 54%, which was higher compared with a recent Italian study [3]. It was not possible to precisely estimate if these symptoms preceded or followed those that prompted medical referral. However, anosmia and dysgeusia should be considered as possible clinical indication of COVID-19 and lead to further investigation including testing for infection with severe acute respiratory syndrome coronavirus 2 (SARS CoV-2) [4].

Comparing admission characteristics between ICU and non-ICU cases, we found that COPD and Horowitz index at the time of hospitalisation were associated with ICU transfer in our series of COVID-19 cases (Table 1). The median days from the onset of symptoms to nasal swab was shorter for cases transferred to ICU probably because of a more severe clinical picture. Concerning laboratory results, we did not notice any difference at admission in IL-6 levels between the two groups, leading us to speculate that the included patients were in an early stage of the disease and IL-6 production might be triggered in the later stages only [5,6].

The overall proportion of cases transferred to ICU described here is higher compared with a Chinese cohort [7]. However, in our preliminary analysis, we observed a 12% reduction in ICU transfer rate adjusted for comorbidities between the pre- and post- intervention period (p = 0.002). This benefit is represented by avoiding endotracheal intubation, taking out possible infectious risks. However, endotracheal intubation should not be delayed in patients with haemodynamic instability, multiorgan failure, or abnormal mental status [8]. Unfortunately, we could not now evaluate if the intervention had an impact also on mortality, because 19 (22.6%) patients were still hospitalised, and mortality rate could have been underestimated in N-ICU. Current evidence against corticosteroid application in COVID-19 is unclear, but the Chinese Thoracic Society recently developed an expert consensus statement on the use of corticosteroids SARS-CoV-2 pneumonia [9]. The increase in corticosteroid prescriptions in the post-intervention period could have played a role in reducing ICU transmission rate. Because of the small sample size and limited outcome information, we could not run a solid multivariable analysis and these results should be cautiously interpreted.

In conclusion, in this Italian cohort, the majority were men older than 60 years with at least one comorbidity in nearly 60%. A self-reported anosmia and/or dysgeusia were frequent symptoms similar to what has been described in a recent European and Italian study [3,4]. The rising intensity of care seemed to reduce the ICU transfer rate and endotracheal intubation. The main limitation of the this study is the small sample size analysed.

## COCORA working group members not listed as individual author

Irene Campolmi, Nicoletta Di Lauria, Giovanni Millotti, Gregorio Basile, Massimo Meli, Pier Giorgio Rogasi, Dario Bartolozzi, Paola Corsi, Marcello Mazzetti, Alberto Farese, Silvia Bresci, Annalisa Cavallo, Michele Trotta, Giampaolo Corti, Alessandro Morettini, Loredana Poggesi, Angelo Raffaele De Gaudio, Adriano Peris, Paolo Fontanari, Paola Parronchi, Fabio Almerigogna, Francesco Annunziato, Francesco Liotta, Lorenzo Cosmi, Alessandra Vultaggio, Andrea Matucci, Manuela Angileri, Alessandra Ipponi, Michele Cecchi, Silvia Benemei, Alessandro Maria Vannucchi, Marco Matucci-Cerinic, Lucia Turco.
