# Authors’ correction for Euro Surveill. 2020;25(17)

**DOI:** 10.2807/1560-7917.ES.2020.25.19.20200514c

**Published:** 2020-05-14

**Authors:** Eurosurveillance editorial team

**Affiliations:** 1 European Centre for Disease Prevention and Control (ECDC), Stockholm, Sweden

In the article ‘Early experience of an infectious and tropical diseases unit during the coronavirus disease (COVID-19) pandemic, Florence, Italy, February to March 2020’ by Lagi et al, published on 30 April 2020, the following corrections were made on 11 May 2020 on request of the authors: the number of patients discharged at the cut-off date was corrected. Furthermore, in Table 1, numbers for ‘High Flow with Nasal Cannula > 24h’ were corrected and under ‘Clinical and laboratory characteristics’, the number of patients for whom information on IL-6 (pg/mL) results was available was wrong in the text and in Table 2 and corrected. The corrections referring to IL-6 (pg/mL) serum levels resulted in a statistically significant difference, where there was no difference in the original publication, between ‘Not ICU transferred’ and ‘ICU transferred’ patients. Consequently, one of the conclusions was changed from ‘Concerning laboratory results, we did NOT notice any difference at admission in IL-6 levels between the two groups, leading us to speculate that the included patients were in an early stage of the disease and IL-6 production might be triggered in the later stages only [5,6].’ to ‘Concerning laboratory results, we noticed a difference at admission in C-reactive protein and IL-6 levels between the two groups, leading us to speculate that those parameters could be early markers for ICU transfer [5,6].’ Together with these corrections, footnotes were added to Tables 1,2 and 4 to indicate variables for which Kruskal-Wallis test was applied.

The authors apologise for the inconvenience.

